# Telehealth Competency Questionnaire-Consumer: Psychometric Validation of a Client-centered Measure

**DOI:** 10.5195/ijt.2023.6598

**Published:** 2023-12-12

**Authors:** Steven Taylor, Lauren Little

**Affiliations:** 1 Department of Occupational Therapy, College of Health Sciences, Rush University, Chicago, IL, USA

**Keywords:** Psychometric validation, Questionnaire development, Telehealth competency, Telehealth consumer

## Abstract

To effectively access telehealth services, individuals must possess certain competencies; yet, telehealth consumer focused measures are limited. The purpose of this study was to describe the development and validation of the Telehealth Competency Questionnaire – Consumer (TCQ-C). Among a sample of adults with chronic health conditions (n=134), findings showed that the TCQ-C is comprised of one factor that accounts for 66.6% of the variance, and internal consistency of subscales are good (range α = 0.80–0.87) and may be used for clinical or research purposes. The TCQ-C demonstrated moderate concurrent validity with the Telehealth Usability Questionnaire-Usability subscale (r = 0.728, p<.001), and significantly discriminates between adults >65 years and those younger as well as those with and without previous telehealth experience. The TCQ-C is a psychometrically sound instrument to evaluate baseline competencies among telehealth consumers so that education, research, and clinical practices are tailored to increase effective engagement between clients and providers.

For over two decades, telehealth has offered the potential to increase access to healthcare services; yet, persistent disparities have spanned this time frame, creating inequities in care. In a 2001 report to the United States Congress, the Department of Health and Human Services (U.S. HHS) noted, “Telemedicine can greatly increase access, but it also has the potential to act as a barrier… the ‘digital divide’ separating those, who have access to computers and the Internet, and those who do not. Will there be a similar digital divide for those seeking health care in the future?” (p. 51). At that time, Medicare reimbursement for specific telehealth services had been in place for two years, though only 301 claims were filed over this period (U.S. HHS, 2001). Approximately twenty years later, utilization of telehealth has grown exponentially to account for 7.0% of all insurance claims in the United States (FAIR Health, 2021). Unfortunately, the digital divide predicted two decades prior has also grown exponentially.

While the majority of individuals within the United States have broadband or Internet enabled smart devices, many inequities remain among several groups to this access (Vogels, 2021). According to the Pew Research Center, 25% of adults 65 years of age and older do not access the Internet, and only 44% own a smartphone or tablet computer (Faverio, 2022). Among individuals whose household income is less than $30,000, 25% do not own a smartphone and 13% have no technologies to access the Internet at home (Vogels, 2021). For these populations of older adults with low incomes, healthcare access via telehealth is impeded due to systemic and compounding barriers (Husain et al., 2022). The intersectionality among mutually reinforcing barriers to access must be better understood to promote equity with healthcare access (Husain et al., 2022).

The digital divide is not limited to internet and device access but instead encompasses the skills needed to operate and engage with technology to successfully participate in a telehealth visit. Although proper skills and training are needed for consumers to effectively engage in telehealth encounters (e.g., how to set up your environment for an encounter; how to troubleshoot technology difficulties), there is little consensus in the literature about what specific skills are needed. The majority of research is focused on provider competencies, yet telehealth is a reciprocal health encounter. Therefore, the *National Academic Consortium of Telehealth* (NACT) outlined practice recommendations for both telehealth providers and users (Hollander et al., 2018), which include technical skills, team-based care, communication, and virtual rapport. It is clear that providers must possess competencies related to the abovementioned skills, yet we have no existing measure of how consumers may perceive their own competence. To create effective education programs for consumers to more effectively use telehealth services, we must first be able to reliably capture their perceptions of competence.

To this end, researchers have developed measures to assess specific aspects of consumer experiences of telehealth. In a recent review of consumer-focused questionnaires used to evaluate telehealth services, Hajesmaeel-Gohari and Bahaadinbeigy (2021) found that the most commonly used measures evaluate satisfaction, usability, acceptance, and implementation. Authors concluded that a questionnaire with few questions and more comprehensiveness is needed. Therefore, the purpose of this article is to describe the development and psychometric validation of the *Telehealth Competency Questionnaire – Consumer* (TCQ-C). Specific research questions included: (1) What is the factor structure of the TCQ-C?; (2) What is the internal consistency of proposed subscales of the TCQ-C?; (3) What is the concurrent validity of the TCQ-C with the Telehealth Usability Questionnaire? (Parmanto et al., 2016); and (4) What is the discriminant validity of the TCQ-C based on age and previous use of telehealth?

## Method

### Measure Development

We developed the TCQ-C using a 5-point Likert scale (1=highly agree to 5=highly disagree) following the guidelines of DeVellis and Thorpe (2021). To guide item development, we consulted research, existing measures, and recommendations about the knowledge, skills, and attitudes (i.e., competencies) necessary to engage in telehealth (e.g., Association of American Medical Colleges [AAMC], 2021; American Academy of Ambulatory Care Nursing [AAACN], 2018; van Houwelingen et al., 2016). After a review of recurrent themes among available literature, we outlined five areas of competency as most relevant for telehealth consumers (see Table 1).

**Table 1.** Telehealth Consumer Competency Domains

Domain	Definition (*Knowledge, skills and attitudes of/towards*…)	References Supporting Competency Domain
Telehealth Usability Fundamentals	Telehealth concepts; considerations for use; safety issues; privacy; client rights	AAACN, 2011; AAMC, 2021; Hollander et al., 2018; Luxton et al., 2014; van Houwelingen et al., 2016
Troubleshooting	Telehealth hardware and software problem solving; requesting accommodations	AAMC, 2021; Hollander et al., 2018; Luxton et al., 2014; van Houwelingen et al., 2016
Virtual Rapport	Environmental and in-session strategies to develop rapport through telehealth; self-advocacy	AAACN, 2011; AAMC, 2021; Agha et al., 2009; Elliott et al., 2020; Henry et al., 2017; Hollander et al., 2018; Luxton et al., 2014; van Houwelingen et al., 2016
Care Planning	Virtual assessment types; shared-decision making; post-session follow-up	AAACN, 2011; AAMC, 2021; Elliott et al., 2020; Hollander et al., 2018; Luxton et al., 2014; van Houwelingen et al., 2016
Participation in Team-Based Care	Telehealth providers, roles and responsibilities; effective communication; team-based decision making	AAACN, 2011; Hollander et al., 2018; Milani et al., 2014

After competency areas were defined, the authors developed a preliminary list of 15 items. An interdisciplinary team of healthcare providers (n=5), identified as content experts in telehealth at Rush University Medical Center, reviewed the list of competencies. Based on feedback, the authors refined the list of competencies, reduced the number of items to 13, and revised language to ensure a 7^th^ grade reading level to align with average-difficulty health literacy accessibility recommendations from the U.S. Department of Health and Human Services (2010).

### Participants

We electronically administered the TCQ-C to a convenience sample of adults with one or more chronic health conditions (n=134) via REDCap (Harris et al., 2019). We recruited the sample from a university registry of older adults that participate in interprofessional education activities. To be eligible, participants needed to be 18 years or older, community dwelling, and have one or more chronic health condition. We included those with chronic health conditions, as they would necessitate telehealth visits beyond preventative care and often interact with a team of healthcare professionals. Demographics of our sample are provided in Table 2.

**Table 2.** Participant Demographics

	n (%)
	Mean = 64.72 years (SD = 12.852)
Age, years	Range = 22 – 88 years
Annual Median Household Income of Zip	
15,000 – 34,999	15 (11.2)
35,000 – 49,999	33 (24.6)
50,000 – 74,999	36 (26.9)
75,000 – 99,999	22 (16.4)
100,000+	27 (20.9)
Race:	
American Indian or Alaskan Native	1 (0.7)
Asian	4 (3.0)
Black or African American	54 (40.3)
White	54 (40.3)
Other	1 (0.7)
Multiracial	2 (1.5)
Unknown	2 (1.5)
Prefer not to report	16 (11.9)
Ethnicity:	
Hispanic or Latino	15 (11.2)
Not Hispanic or Latino	103 (76.9)
Prefer not to report	16 (11.9)
Previous use of Telehealth:	
Yes	64 (47.8)
No	70 (52.2)

### Data Analysis

We used SPSS 28.0 to analyze data. To address the factor structure of the TCQ-C, we used principal components analysis (PCA). To determine the number of factors, we considered eigenvalues over 1.0 and item loadings (Guttman, 1954). We considered items with loadings >.32 (Tabachnick et al., 2013) and considered items as cross-loading if they loaded within 0.1 on more than one factor (Ferguson & Cox, 1993). To address the internal consistency of proposed TCQ-C, we used Cronbach's Alpha, and we used Pearson correlations to evaluate the concurrent validity of the TCQ-C with the TUQ Usefulness subscale, which has good internal consistency (standardized α = 0.85) (Parmanto et al., 2016). Lastly, we used t-tests to determine discriminant validity between adults <65 years and >65 years as well as those that had previously used telehealth versus those that had not.

## Results

**Figure 1.**
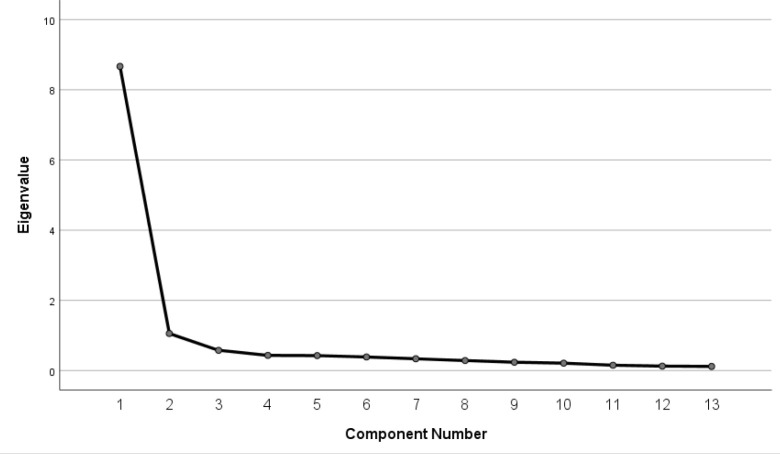
Principal Component Analysis Scree Plot

*Factor structure of the TCQ-C.* We ran principal component analysis (PCA) on the 13-item TCQ-C questionnaire. Prior to PCA, we assessed suitability of the data for factor analysis and found many coefficients were greater than 0.3. The Kaiser-Meyer-Oklin value was 0.945, exceeding the 0.6 cutoff of Kaiser (1974) and Bartlett's Test of Sphericity (Bartlett, 1954) was significant at *p*>0.001; supporting factorability of the correlation matrix. The analysis revealed the presence of two factors with eigenvalues exceeding 1.0; however, inspection of the scree plot revealed a clear break after the first component (See Figure 1), with the second factor accounting for only 8.6% of the variance.

Parallel analysis confirmed one component with eigenvalues exceeding the criterion values from a randomly generated data matrix of the same size (13 items, 134 participants). The factor solution explained a total of 66.7% of the variance. We performed Oblim rotation to aid in the interpretation of the components. The rotated solution resulted in strong item loadings on one factor, and no item cross-loaded on two factors >0.3. Range of item loadings was 0.71–0.88 (see Table 3).

**Table 3.** Telehealth Competency Questionnaire – Consumer (TCQ-C) Factor Loadings and Subscale Internal Consistency

TCQ-C Item	Factor Loading	Mean (SD)
**Telehealth Usability Fundamentals Subscale**		
I have knowledge of how to use Telehealth services	0.785	2.57 (1.166)
I have knowledge of what to do in case of an emergency when using of Telehealth.	0.716	3.06 (1.314)
I feel my privacy is secure when using Telehealth.	0.720	2.41 (1.063)
**Troubleshooting Subscale**		
I can problem-solve steps to take if Telehealth technology does not work.	0.777	2.66 (1.137)
I have knowledge of ways Telehealth can be adapted to meet my needs.	0.875	2.71 (1.149)
**Care Planning Subscale**		
I have knowledge of the process of Telehealth care planning.	0.875	2.85 (1.173)
I feel I can have a shared role in healthcare decisions through Telehealth.	0.838	2.21 (0.989)
I can make my needs be known to care providers when using Telehealth.	0.866	2.02 (0.862)
**Virtual Rapport Subscale**		
I feel a therapeutic (caring) relationship with my Telehealth care providers is of value.	0.752	2.02 (0.905)
I have knowledge of how to set up my surroundings for a Telehealth session.	0.858	2.57 (1.172)
I can communicate clearly when using Telehealth.	0.880	2.26 (0.996)
**Participation in Team-based Care Subscale**		
I can work together with multiple care providers through Telehealth.	0.808	2.31 (0.969)
I feel I can be an active member of my healthcare team through Telehealth.	0.839	2.12 (0.918)

*Note*. The TCQ-C uses a 5-level Likert scale for all items ('1': strongly agree; ‘2’: agree; ‘3’ neutral; ‘4’: disagree; ‘5’: strongly disagree).

*Internal Consistency of TCQ-C Subscales.* While PCA showed a one factor solution, we wanted to investigate the internal consistency of proposed subscales for clinical, education, and research purposes. Results showed the following values: Telehealth Usability Fundamentals subscale α = 0.81; Troubleshooting subscale α = 0.80; Virtual Rapport subscale α = 0.81; Care Planning subscale α = 0.87; and Participation in Team-based Care subscale α = 0.85.

*Concurrent Validity of the TCQ-C.* Pearson product-moment correlation coefficient between the TCQ-C and TUQ Usability subscale showed a moderate, positive correlation between these two variables, r = 0.728, *p*<.001.

*Discriminant Validity of the TCQ-C.* To investigate the discriminant validity of the TCQ-C based on participant age, we divided the sample between those 65 years and older (n=77) versus 64 years and younger (n=57). T-test results showed significant differences between age groups in total TCQ-C score (t= −1.61, 132, *p*<.001), with older adults reporting worse skills and abilities in using telehealth (M=2.82; SD=0.86) as compared to younger adults (M=2.40; SD=.0.82). T-test results showed significant differences between those that had not previously used telehealth (n=70; M=2.20, SD=0.87) versus those that had (n=64; M=2.94, SD=0.68) (t=2.66, 132, *p*<.001).

## Discussion

In alignment with recommendations of NACT, consumer-oriented trainings may be one approach to address inequities in telehealth access. However, it is first necessary to understand perceptions of telehealth competency where disparities presently exist, and how disparities may be influenced by the intersectionality of systemic barriers. Therefore, the purpose of this study was to describe the development of a measure assessing telehealth consumers' perceptions of competency (i.e., the TCQ-C). If we can reliably capture the areas in which consumers report variable levels of competency, we can design education, clinical, and research programs to best target their needs.

The TCQ-C was comprised of one factor, which we describe as overall consumer telehealth competency. All items loaded within the moderate to high range on one factor, and when we investigated the two-factor solution, no items loaded within 0.3 within one another, offering further support for the one factor solution. While the measure shows evidence of one factor, we did find high internal consistency values for the proposed subscales of the TCQ-C, which include Telehealth Usability Fundamentals, Troubleshooting, Virtual Rapport, Care Planning, and Participation in Team-based Care. We argue that these subscales may be important clinically, so that a full range of skills associated with effective telehealth access may be assessed.

The majority of consumer-focused measures of telehealth assess satisfaction, usability, and acceptance (Hajesmaeel-Gohari & Bahaadinbeigy, 2021). Therefore, the TCQ-C provides a more comprehensive picture of the range of skills needed to access telehealth. While there are fundamental skills needed for a consumer to participate in telehealth sessions (e.g., logging on to the virtual platform), the TCQ-C also captures the interpersonal skills needed for those with chronic conditions to effectively manage their care, such as being an active member of their healthcare team and communicating with multiple healthcare providers.

Lastly, the TCQ-C showed moderate concurrent validity with the TUQ-Usefulness Subscale, which we would expect given that the TCQ-C assesses a wider range of behaviors and perceptions associated with telehealth use. The TCQ-C also demonstrated the ability to discriminate between adults >65 years and those younger and those with and without previous telehealth experience. Given that the digital divide disproportionately impacts older adults (Faverio, 2022), such evidence of discriminant validity shows that the TCQ-C may be particularly helpful in assessing consumer baseline skills to develop tailored training programs for specific needs and specific individuals.

## Limitations

The current study presents with limitations. First, the TCQ-C represents a consumer's perception of their competencies and may not align with objective or observational measures of skills demonstrated by a consumer during a telehealth encounter. Second, as telehealth continues to expand and new virtual platforms are developed, consumers may become increasingly competent in accessing services or new areas of competency may arise. Lastly, our sample was limited to those with chronic conditions in one geographical area and samples of those without chronic health conditions or from other geographical areas may present with different competencies and/or needs within telehealth encounters.

## Conclusion

Telehealth demonstrates potential to increase access to health services where disparities exist, yet consumer-oriented training is necessary to promote equity among prospective users. The TCQ-C is a measure with demonstrated validity to assess user perceived telehealth competency. It may be used to increase our understanding of the unique telehealth training needs among populations for whom perception of competency is a barrier.
